# Embryonic Signaling Pathways and Rhabdomyosarcoma: Contributions to Cancer Development and Opportunities for Therapeutic Targeting

**DOI:** 10.1155/2012/406239

**Published:** 2012-04-30

**Authors:** Brian Belyea, Julie Grondin Kephart, Jordan Blum, David G. Kirsch, Corinne M. Linardic

**Affiliations:** ^1^Department of Pediatrics, Duke University Medical Center, Durham, NC 27710, USA; ^2^Department of Pediatrics, University of Virginia, Charlottesville, VA 22908, USA; ^3^Department of Pharmacology & Cancer Biology, Duke University Medical Center, Durham, NC 27710, USA; ^4^Department of Radiation Oncology, Duke University Medical Center, Durham, NC 27710, USA

## Abstract

Rhabdomyosarcoma is the most common soft tissue sarcoma of childhood and adolescence, accounting for approximately 7% of childhood cancers. Current therapies include nonspecific cytotoxic chemotherapy regimens, radiation therapy, and surgery; however, these multimodality strategies are unsuccessful in the majority of patients with high-risk disease. It is generally believed that these tumors represent arrested or aberrant skeletal muscle development, and, accordingly, developmental signaling pathways critical to myogenesis such as Notch, WNT, and Hedgehog may represent new therapeutic targets. In this paper, we summarize the current preclinical studies linking these embryonic pathways to rhabdomyosarcoma tumorigenesis and provide support for the investigation of targeted therapies in this embryonic cancer.

## 1. Introduction

Rhabdomyosarcoma (RMS) is the most common soft tissue sarcoma of childhood and adolescence, accounting for approximately 7% of all pediatric cancers [1, 2]. Tumors are classified based on their histology as embryonal (~60%), alveolar (~20%), or other (~20%) subtypes. Embryonal (eRMS) and alveolar (aRMS) subtypes have unique genetics, patterns of involvement, biology, and prognosis. Approximately 80% of aRMSs contain a stable reciprocal translocation involving either the PAX3 or PAX7 transcription factors with the FOXO1 transcription factor, resulting in a fusion gene with altered transcriptional and translational properties, while 80% of eRMSs have loss of heterozygosity in chromosome 11p15 [1, 3]. aRMS more commonly occurs in the extremities and has a high predilection for metastasis, while eRMS is more likely to present as localized disease in the genitourinary or head/neck regions. Patients with aRMS have an inferior survival rate compared to embryonal subtype, even when controlled for risk groups, with eRMS and aRMS having five-year OS of 80% and 52%, respectively, [4]. In addition to histologic subtype, patients are risk-stratified based on pretreatment stage and clinical group. Currently, the Children's Oncology Group (COG) Soft Tissue Sarcoma committee (formerly Intergroup Rhabdomyosarcoma Study Group (IRSG)) classifies and treats patients according to low, intermediate, and high risk prognostic groups.

The current treatment strategies for RMS include chemotherapy, radiation, and surgery. Over the last 50 years, the survival rates for patients with low- and intermediate-risk disease have improved significantly due to risk stratification, combination chemotherapy regimens, and cooperative clinical trials [1]. The IRSG was formed in 1972 and performed five successive clinical trials over the next 25 years. Subsequently, the Soft Tissue Sarcoma (STS) committee of the COG has continued to conduct clinical trials for RMS. During this time, the survival rates for patients with low- and intermediate-risk disease improved significantly from a ~25% OS to the most recent studies with mature data for low-risk (IRSG IV) and intermediate-risk RMS (D9803) demonstrating a five-year FFS of 90% and four-year OS of 71%, respectively, [5, 6]. Similarly, multiple international cooperative groups including the Italian Group, International Society of Pediatric Oncology (SIOP), CWS, and European Intergroup have demonstrated improvements in the survival for patients with localized disease [7]. Unfortunately, the survival rate for patients with high-risk disease has not similarly improved above the dismal 20–30% rate [7]. The most recent mature study of patients with high-risk RMS enrolled in COG studies (IRS IV) reported a three-year OS of 5% for the highest-risk group (>three metastatic sites, unfavorable histology) [8]. In addition, two consecutive European Intergroup Studies of childhood metastatic RMS (MMT4-89 and MMT4-91) also demonstrated persistently poor outcomes with a five-year overall survival rate of 24% [9]. Finally, patients with refractory and relapsed disease continue to do very poorly with an OS of 10% at five years [10]. Recent efforts to improve this poor prognosis in high-risk patients have included multiagent, interval compressed therapy, high-dose chemotherapy with stem cell rescue, oral maintenance regimens, and novel targeted therapies including growth factor inhibition and regulation of angiogenesis. While these current studies offer hope for improved cure rates in high-risk RMS, there remains a great need for new therapies targeting the molecular pathways that are involved in the pathogenesis of RMS. We believe that strategies aimed at reducing the regenerative potential of tumors by inhibiting the embryonic pathways critical to tumor initiation and maintenance will be important for future investigation.

RMS is believed to arise from skeletal muscle precursors that fail to undergo appropriate terminal differentiation. Although the precise cell of origin is unclear, skeletal muscle precursors are obvious suspects because they already possess “stemness” characteristics—features that are shared with cancer cells—such as self-renewal, high rates of proliferation, resistance to senescence, and reversal of quiescence. In addition to these stem cell properties, skeletal muscle precursors share stemness markers with RMS cancer cells including PAX3, PAX7, MET, and myogenic regulatory factors [11, 12]. In this scenario, a single somatic mutation that blocks the differentiation capacity of muscle precursors might be sufficient to cause malignant transformation. Alternatively, such a mutation may arrest normal differentiation and thus expand the number of muscle precursors “at risk” for additional transforming events. Finally, inappropriate embryonic cell signaling after cells have previously differentiated beyond a stem cell fate may reactivate the molecular mechanisms necessary for dedifferentiation phenotypes and stemness behaviors, resulting in tumorigenesis [13]. In skeletal muscle, the Notch, WNT, and Hedgehog signaling pathways regulate self-renewal versus differentiation fates of muscle stem cells and thus control the balance of proliferation versus differentiation. These embryonic signaling pathways that are responsible for normal skeletal muscle differentiation may therefore play a critical role in RMS tumorigenesis by stabilizing muscle precursor cells and allowing more opportunities for secondary, cooperating mutations to occur, by directly inhibiting normal differentiation leading to transformation or by promoting dedifferentiation of mature muscle cells. In this paper, we review the physiologic role of several of these embryonic signaling pathways including Notch, WNT, and Hedgehog in myogenesis as well as the preclinical data that supports their role in RMS tumorigenesis.

## 2. Skeletal Myogenesis

Myogenesis is a complex and carefully timed process that results in discrete muscle groups that are fully functional even shortly after birth. In mammals, skeletal myogenesis begins during embryonic life and proceeds through fetal, neonatal, and adult developmental stages [14]. There are three ways in which embryonic myogenesis can be described: developmental stage, phase of myogenesis, or type of myoblast [14–16]. Fortunately, these different ways of describing myogenesis parallel each other except for the neonatal developmental stage as there is no known myoblast unique to it [14]. Myogenesis is initiated when the presomitic paraxial mesoderm contracts to form somites, which then differentiate into the dermomyotome and the sclerotome [17]. The epaxial and hypaxial myotomes are formed from the dermomyotome, the source of all trunk and limb muscles. Cells that will form muscle in the limbs undergo an epithelial to mesenchymal transition, delaminate from the dermomyotomes, and migrate to their final tissue locations [17]. During embryonic myogenesis, or the first myogenic phase, the primary myotome is developed and the basic muscle pattern established. In the limb, this occurs between E10.5 and E12.5 in mice [14, 15]. Both fetal myogenesis (in the limb, E14.5-P) and neonatal (P0-21) myogenesis are encompassed by the second myogenic phase, which generates the adult musculature and allows for growth and development of muscle. Finally, adult myogenesis (after P21) occurs through the use of satellite cells and is responsible for postnatal growth and repair. Further detailed information about myogenesis can be found in [14–17].

### 2.1. Embryonic Signaling Pathways and Skeletal Myogenesis

Embryonic signaling pathways including Notch, WNT, and Hedgehog play a critical role in the transition of muscle precursors from self-renewal to differentiation fates during both muscle development and postnatal muscle regeneration. During muscle development and postnatal muscle regeneration, mononucleated progenitor cells undergo amplification and asymmetric differentiation along a myogenic lineage to form multinucleated myotubes and ultimately myofibers, which are the terminally differentiated unit of skeletal muscle. In both fetal and postnatal development, there remains a small population of mononuclear muscle stem cells (fetal) or satellite cells (postnatal) that remain undifferentiated and retain the ability to proliferate and differentiate in response to growth signals or tissue damage. In normal myogenesis, the Notch, WNT, and Hedgehog signaling pathways have been shown to regulate the progression of muscle stem cells towards lineage-committed progenitors, and, therefore, these embryonic signaling pathways function to preserve and expand this subpopulation of muscle stem cells. Given the critical role of these pathways in myogenesis, aberrant expression, activation, or regulation of these pathways may be involved in RMS tumorigenesis and may present novel therapeutic targets in the treatment of RMS. Specifically, RMS may exploit the function of these pathways in order to allow for persistent stem cell maintenance, self-renewal, and tumor initiation (Table 1).

### 2.2. Notch Pathways

**Figure 1:**
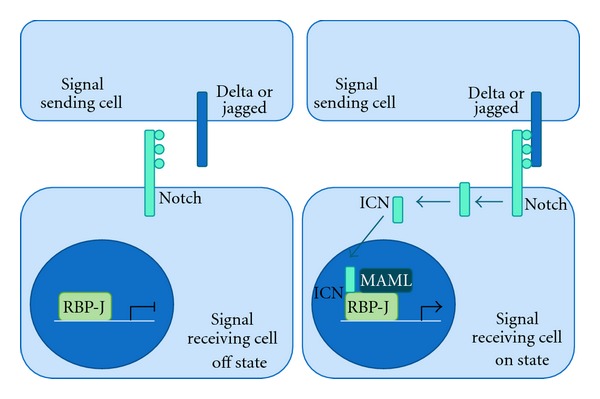
Overview of the Notch pathway. The pathway is active when a Notch ligand, such as Delta or Jagged, binds to a Notch receptor on a neighboring cell. Notch is cleaved and the cytoplasmic portion (ICN) translocates to the nucleus where it binds RBP-J in cooperation with Mastermind (MAML) to activate transcription of target genes.

 The Notch signaling pathway is an evolutionarily conserved signaling pathway that mediates many tissue progenitor cell fate decisions including the determination between self-renewal and differentiation. This pathway includes multiple transmembrane receptors and ligands that function through direct cell surface contact. In mammals, there are four Notch receptors (Notch1–4) and five Notch ligands (Jagged1,2 and DLL1,3,4). Signaling is initiated when neighboring cells come into direct contact resulting in ligand-receptor binding. Activation of the receptor by ligand binding is followed by a two-step proteolytic cleavage of the cytoplasmic portion of the receptor and release of the cleaved active Notch receptor (intracellular Notch, ICN). ICN then translocates to the nucleus where it binds to the transcription factor RBP-J in cooperation with the coactivator Mastermind, resulting in a large transcriptional activation complex that promotes the transcription of target genes (Figure 1). In muscle stem cells, Notch regulates the balance between maintenance of progenitor cells by inhibition of differentiation versus the facilitation of commitment to muscle lineage.

Notch signaling maintains muscle progenitors during both embryogenesis and postnatal development by suppressing myogenic differentiation. Studies in the 1990s demonstrated that overexpression of ICN in murine myoblasts inhibits their differentiation [25], with subsequent studies demonstrating multiple mechanisms for this differentiation block. Kuroda et al. demonstrated that activation of the Notch signaling pathway via overexpression of Notch ligands results in inhibition of muscle regulatory factors including MyoD in mouse myoblasts [26]. In addition, activation of the Notch pathway reduces the transcription of the promyogenic genes myogenin and MEF2c by the Notch target gene Hey1 [27], and, furthermore, ICN directly inhibits the transcriptional activity of MEF2c [28].

In addition to inhibition of differentiation, Notch signaling promotes the renewal of stem cells during embryogenesis and satellite cells during postnatal myogenesis. Over-expression of activated Notch (ICN) in myoblasts results in proliferation of satellite cells and inhibition of differentiation into fusion-competent myoblasts [29]. In agreement with these results, Notch signaling loss-of-function studies have demonstrated inappropriate myogenic differentiation in muscle progenitors. While there is redundancy in the Notch ligands and receptors, the transcription factor RBP-J is the central nuclear mediator of Notch signaling. Using a conditional knockout of RBP-J in mouse muscle precursor cells, Vasyutina et al. showed that inactivation of the Notch pathway results in inappropriate differentiation of myogenic cells and depletion of muscle progenitor cells during embryogenesis [30]. Similarly, in postnatal muscle repair following injury, inhibition of the Notch pathway reduces the ability of satellite cells to regenerate muscle [29]. In conclusion, the Notch signaling pathway promotes muscle stem cell maintenance by inhibiting early differentiation and expanding pools of progenitor cells during both embryogenesis and postnatal muscle regeneration; however, it does not appear to be involved in the direction of terminally differentiated cells.

### 2.3. Wnt Pathway

**Figure 2:**
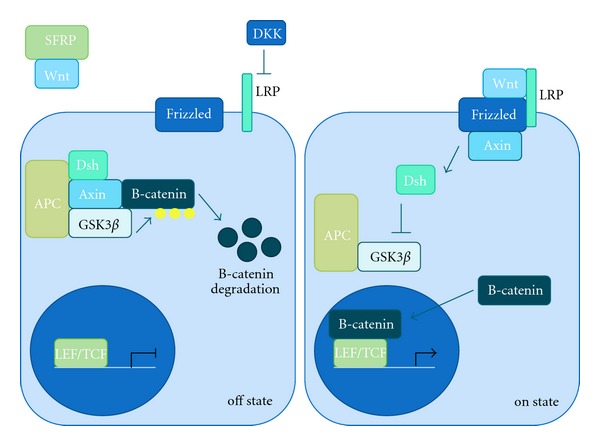
Overview of the canonical Wnt pathway. In the inactive state, Wnt is absent and *β*-catenin is phosphorylated by the destruction complex, leading to its degradation. In the active state, Wnt binds Frizzled (Fzd) and LRP. Dishevelled (Dsh) and axin are recruited to the Wnt-Fzd-LRP complex, which inhibits the destruction complex. *β*-catenin is no longer phosphorylated and can translocate to the nucleus to activate transcription of target genes.

 Similar to Notch, the WNT signaling pathway is conserved in all animals, including worms, flies, and mammals [31]. In mammals, Wnt signaling is divided into a number of pathways, including the canonical/*β*-catenin, the noncanonical planar cell polarity (PCP), and the Wnt/Ca^2+^ pathways [32]. Of these, the canonical/*β*-catenin pathway is the best understood. In the absence of Wnt, the pathway is inactive, allowing for the phosphorylation of *β*-catenin by glycogen synthase kinase 3 (GSK3) and casein kinase 1 (CK1). These two kinases along with scaffolding proteins Axin and adenomatous polyposis coli gene product (APC) form the destruction complex. Once *β*-catenin is phosphorylated, it is recognized and targeted for proteasomal degradation by *β*-Trcp, an E3 ubiquitin ligase [31]. Alternatively, in the presence of Wnt, the pathway is active. Wnt binds the extracellular region of Frizzled (Fzd) and its coreceptor low-density lipoprotein receptor-related protein 6 (LRP6) or LRP5. The formation of a Wnt-Fzd-LRP complex recruits Dishevelled (Dsh) and results in the recruitment of Axin and the rest of the destruction complex. This leads to inhibition of the destruction complex, allowing for stabilization of *β*-catenin, which accumulates in the cytoplasm and translocate to the nucleus where it forms complexes with TCF/LEF and activates Wnt target genes (Figure 2) [31]. Inhibitors of the Wnt pathway include the secreted frizzled-related protein (SFRP) family and Wnt inhibitory proteins (WIF), which inhibit Wnt directly via direct binding, and the Dickkopf (DKK) family, which inhibit LRPs [31].

During skeletal myogenesis, Wnt has various roles in embryonic, fetal, and neonatal myogenesis. At the onset of embryonic myogenesis, MyoD is activated in the presomitic mesoderm by Wnt7a signaling through a *β*-catenin independent pathway [33]. In contrast, Myf5 expression in somites is dependent on *β*-catenin signaling, and expression of *β*-catenin is sufficient to induce Myf5 expression in the somites [34]. Interestingly, in the presomitic mesoderm, both *β*-catenin signaling and sonic hedgehog/Gli signaling are required for Myf5 expression [34]. Further, Wnt1 preferentially activates Myf5, and Wnt7a preferentially activates MyoD in the presomitic mesoderm; however, Wnt4, Wnt5a, and Wnt6 can moderately activate both MyoD and Myf5 expression [35]. During embryonic myogenesis, *β*-catenin is required for dermomyotome and myotome formation, but not required for embryonic axial myogenesis [36]. Fetal myogenesis, consistent with having different myogenic progenitors than embryonic myogenesis, has different requirements for *β*-catenin. In contrast to embryonic myogenesis, *β*-catenin is important for fetal limb myogenesis, particularly the formation of slow fibers. Further, *β*-catenin positively regulates the number of Pax7+ myogenic progenitors [36].

 In adult myogenesis, the role of the Wnt pathway and *β*-catenin is unclear, as some data demonstrate that activated *β*-catenin promotes myogenic lineage progression and differentiation, while other studies find that it promotes satellite cell proliferation and inhibits differentiation. In regenerating muscle, activating or inhibiting the Wnt pathway early (two days) following injury did not alter myogenesis; however, activating the Wnt pathway late (four days) following injury increased myogenic differentiation [37]. Further, a transition from Notch to Wnt signaling is important for the progressing along the myogenic lineage from quiescent satellite cell to fully differentiated muscle [37]. In CD45+ stem cells, activation of the Wnt pathway allows these cells to enter the myogenic lineage and undergo myogenic differentiation [38]. This myogenic differentiation can by blocked by inhibition of the Wnt pathway using SFRPs in satellite cells and CD45+ stem cells [37–39]. Interestingly, increased Wnt signaling in the myogenic progenitor cells of aged mice (~24 months) leads to their conversion from a myogenic to fibrogenic lineage and contribute to tissue fibrosis [40]. Finally, growth of muscle (hypertrophy) following muscle overload appears to require *β*-catenin expression [41].

In contrast, *β*-catenin increased proliferation of Pax7+ satellite cells and inhibited their differentiation in both an *in vitro* and an *in vivo* setting [42, 43]. Further, demonstrating the complexity of this pathway in regulating myogenesis, only Wnt1, Wnt3a, and Wnt5a promoted satellite cell proliferation, while Wnt4 and Wnt6 were inhibitory [42]. Finally, during muscle regeneration Wnt1, Wnt3a, Wnt7a, and Wnt11, the Wnts often associated with embryonic myogenesis, are not induced, but SFPR1, SFRP2, and SFRP3, antagonists of the Wnt pathway, are induced, suggesting a downregulation of the pathway [44]. Future research should aim to reconcile these two seemingly opposite roles of the Wnt pathway and *β*-catenin in myogenic differentiation.

### 2.4. Hedgehog Pathway

The Hedgehog signaling pathway has been rightly described as an enigmatic developmental pathway in metazoans, due in part to an incomplete understanding of how the pathway constituent proteins interact [45]. Currently, it is clear that the Hedgehog signaling cascade is initiated when a Hedgehog protein, such as Sonic Hedgehog (SHH), Desert Hedgehog (DHH), or India Hedgehog (IHH) is translated and subsequently autocleaved, forming both amino-terminal and carboxyl-terminal peptides [46]. The amino-terminal peptide is then transported through the plasma membrane of the signaling cell via the multipass transmembrane protein Dispatched and diffuses across the extracellular space in a signaling gradient [47].

**Figure 3:**
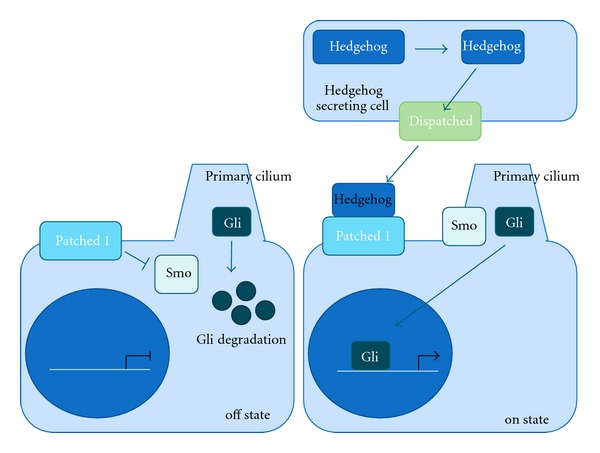
Overview of the Hedgehog pathway. In the inactive state, Patched 1 (PTCH1) inhibits Smoothened (SMO) and Gli is targeted for degradation by the proteasome. In the active state, Hedgehog (HH) is translated, cleaved, and transported by Dispatched to the extracellular space. HH is able to bind to PTCH1, which prevents inhibition of SMO and allows it to accumulate at base of the primary cilium. Gli is then stabilized and translocates to the nucleus to activate transcription of target genes.

In *Drosophila melanogaster*, Hedgehog signaling is mediated by the transcription factor Cubitus interruptus (Ci). In contrast, the mammalian ortholog to Ci is comprised of the three GLI family members GLI1, GLI2, and GLI3. Under homeostatic conditions, cells with the potential to respond to Hedgehog signaling (including the signaling cell) express the cell surface receptor Patched1 (PTCH1), which constitutively inhibits the membrane protein Smoothened (SMO) [48]. When a Hedgehog ligand binds PTCH1, PTCH1 inhibition of SMO is prevented by a mechanism that remains to be fully characterized and SMO is able to accumulate at the base of the primary cilium (in mammals) [49]. SMO localization to the base of the primary cilium leads to the stabilization of cytoplasmic GLI1/2 and degradation of the repressor GLI3, allowing GLI1/2 to translocate to the nucleus and activate downstream targets of Hedgehog signaling such as HHIP, PTCH1, GLI1, and GLI2 (Figure 3) [50].

Similar to the Notch and Wnt pathways, the Hedgehog pathway has been shown to play a crucial role in the regulation of early myogenesis in vertebrates. Hedgehog ligand diffuses from cells within the notochord and ventral neural tube into the developing somites where Hedgehog signaling is required to maintain MYF5 expression in the dorsomedial lip of the dermomyotome [51]. During embryonic and fetal myogenesis, Hedgehog is required for survival, proliferation, and maintenance of myogenic regulatory factors (MRFs) in developing myoblasts and myotubes in both epaxial and hypaxial muscles, although it is dispensable for the initiation of myogenesis in the hypaxial limb of the mouse [52]. Because Hedgehog signaling plays an important role in survival and proliferation in the developing myotome, it is not surprising that it is indispensable for adult skeletal muscle myogenesis.

 Although postnatal skeletal muscle is largely quiescent, the satellite cell has a regenerative capacity akin to highly proliferative stem cell populations such as the hematopoietic stem cell. Recent data suggests that Hedgehog signaling plays a unique role in adult myogenesis because inhibition of Shh via cyclopamine impaired the activation of Myf5 and MyoD in skeletal muscle progenitors and led to a reduction in the number of activated satellite cells and myoblasts following injury *in vivo*. Furthermore, the addition of Shh to C2C12 myoblasts *in vitro* led to an increase in proliferation [53]. In addition, the pathway has been shown to block the differentiation of myogenic precursor cells into myotubes and maintain satellite cells as self-renewing precursors [54]. This group also demonstrated that Hedgehog signaling inhibits apoptosis in muscle precursors. Other studies have concluded that Hedgehog signaling represses terminal differentiation of myoblasts [55]. Although the role of the Hedgehog pathway in satellite cell biology is in its infancy, it is becoming increasingly clear that Hedgehog plays a role not only in activation of satellite cells, but in the proliferation of myoblasts and the process of terminal skeletal muscle differentiation.

### 2.5. Cross-Talk between the Notch, Wnt, and Hedgehog Pathways

The ability of Notch signaling to maintain muscle stem cells in both fetal and adult muscle likely requires the input of the Wnt and Hedgehog signaling pathways important in tissue differentiation and stem cell biology. Brack et al. demonstrated that coordinated transition from Notch activation to WNT activation is required to promote myogenic lineage progression from satellite cells to fusion-competent myoblasts [37]. They additionally show that in skeletal muscle precursors, aberrant activation of Notch signaling or inactivation of WNT signaling prevents myogenic lineage progression [29, 37]. Thus, progression of skeletal muscle stem cells along a myogenic lineage is controlled by early Notch activation and late WNT activation, and the coordinated transition from Notch activation to WNT activation dictates myogenic lineage progression from muscle stem cells to differentiated myogenic progenitors. Though not well studied in mesenchymal tissues, the interaction between the Wnt and Hedgehog pathways can be mediated through GSK3*β*. Briefly, it has been demonstrated in epithelium that loss of Shh in the hair follicle leads to an increase in GSK3*β*, suggesting that activated Hedgehog signaling can, given the appropriate cellular context, potentiate Wnt signaling activation via *β*-catenin stabilization [56]. These studies demonstrate that the cumulative effect of Notch, WNT, and Hedgehog signaling pathways regulates the progression from muscle progenitor expansion to differentiation phenotypes and ultimately contribute to inhibit embryonal myogenesis.

## 3. Rhabdomyosarcomagenesis

 Traditionally the study of RMS tumorigenesis has focused on genes and proteins that have been found to be dysregulated in human, and later murine models of RMS cell lines and tissues. Thus, there has been intense focus, rightly so, on the classic tumor suppressors Rb and p53, classic oncogenes such as Ras and Myc, a variety of receptor tyrosine kinases such as IGF1R, cMET, and of course the aRMS-specific chromosomal translocations. As such, newer treatment agents that can target these moieties, such as the anti-IGF1R monoclonals and small-molecule RTK inhibitors, have made their way through preclinical studies and are being tested in human clinical trials. However, recently there have been developed classes of drugs that can inhibit the Notch, Wnt, and Hedgehog pathways, and this has likely spurred a renewed interest in understanding the biology of these pathways in RMS.

### 3.1. Notch and Rhabdomyosarcoma

 The first study linking the Notch signaling pathway to RMS investigated the influence of Hes1, a Notch target gene, on reversibility of cellular quiescence. In this work, Sang et al. demonstrated that HES1 mRNA expression was increased in 21 primary RMS tumors and three RMS cell lines (one eRMS and two aRMS) [18]. They then used a dnHes1 construct to evaluate the role of this gene on RMS differentiation. Specifically, they transduced an aRMS cell line, RHJT, with either dnHes1 or empty vector control. RMS cells expressing dnHes1 demonstrated early differentiation and inhibited proliferation. Similarly, treatment of RHJT cells with the pharmacologic Notch inhibitor DAPT also promoted differentiation. From these experiments, they concluded that the transcription factor Hes1, via Notch-dependent signaling pathway, contributes to the initiation and progression of RMS by suppressing irreversible states such as differentiation and senescence.

 A subsequent study by Roma et al. evaluated the contribution of the Notch signaling pathway to RMS mobility and invasiveness [19]. Using quantitative real-time PCR, they evaluated 37 primary tumor samples for expression of the four Notch receptors and two Notch target genes, Hes1 and Hey1. They found increased expression of Notch2 and Notch3 in both eRMS and aRMS compared to adult and fetal skeletal muscle. In addition, they found an increase in Hes1 in eRMS and an increase in Hey1 in both eRMS and aRMS. They then showed that Notch inhibition, through both *γ*-secretase inhibitors and dominant negative MAML1 coactivator construct, decreases RMS cell line mobility and invasiveness *in vitro* as measured by wound healing assays and Matrigel/Transwell invasion assays, respectively.

 Recently we have investigated the role of the Notch-Hey1 pathway inhibition in eRMS [20]. Inhibition of Notch signaling via Notch1 shRNA, Hey1 shRNA, or *γ*-secretase inhibitors *in vitro* resulted in decreased proliferation of eRMS cell lines, and reduction of the Notch target gene Hey1 resulted in an increase in early differentiation. In murine eRMS xenograft models, treatment with either Notch1 shRNA or *γ*-secretase inhibitors inhibited tumor growth, providing preclinical evidence of a role for Notch pathway inhibition in eRMS. The role of Notch signaling in RMS stem cell maintenance has not been specifically addressed, although it appears that Notch upregulation via constitutively active ICN1 enhances the formation of “rhabdospheres” (unpublished data), a recently described source of eRMS cancer stem cells that exhibits upregulated stem cell genes and increased tumorigenicity [57]. Further studies are necessary to evaluate if pharmacologic Notch inhibition decreases the cancer stem cell population of RMS.

### 3.2. Wnt and Rhabdomyosarcoma

The Wnt/*β*-catenin pathway was first identified as protumorigenic when an inactivating mutation in APC was described in human colon cancer [58]. Since then, many human cancers have been shown to harbor changes in the Wnt pathway, resulting in upregulated *β*-catenin activity and target gene expression [32]. On the other hand, inactivation of GSK3 (which activates *β*-catenin) causes cell cycle arrest in leukemia [59], decreased cell proliferation in pancreatic cancer [60], and apoptosis in colorectal cancer [61]. Furthermore, the Wnt pathway inhibitor SFRP3 is upregulated in metastatic renal carcinoma [62]. Thus, while the prevailing view had been that upregulation of the Wnt/*β*-catenin pathway necessarily meant that it was tumorigenic, the role of Wnt pathway members such as GSK3 in other signaling pathways and the cell or tissue context of the Wnt pathway signals suggest that the role of Wnt signaling in tumorigenesis is more complex.

Currently, very little is known about the role of Wnt in RMS. In eRMS, the Wnt pathway was investigated using cells derived from eRMS tumors formed in p53^−/−^/cfos^−/−^ mice and human eRMS cells (RD cells) [21]. These cells overexpress Wnt2 but show downregulated *β*-catenin activity when compared to normal muscle myoblasts. Reactivating the Wnt pathway in eRMS cells induced MyoD expression and promoted differentiation. This work suggests that inducing the Wnt pathway could be a potential therapeutic approach for treating eRMS. Also in a small set of samples from patients' tumors, aRMS, eRMS, and sclerosing RMS (sRMS) all show cytoplasmic localization of *β*-catenin, suggesting that a downregulation of the Wnt pathway is a common feature of RMS tumors; however this downregulation is not due to a mutation in *β*-catenin [63]. This was also observed in a different set of patient tumor samples, in which no RMS contained over 25% of cells staining positive for nuclear *β*-catenin and only 15% of RMS contained any cells staining positive for nuclear *β*-catenin [64].

In aRMS, GSK3 inhibitors, which result in the activation of *β*-catenin, appeared to preferentially inhibit cell proliferation and induce apoptosis when compared with eRMS cells [22]. Further, GSK3 inhibitors appeared to reduce the activity of the PAX3-FOXO1 protein, as PAX3-FOXO1 is phosphorylated by GSK3. Previous work had demonstrated that inhibiting PAX3-FOXO1 activity through the use of a PAX3-KRAB does inhibit growth of aRMS cells in conditions of low serum or soft agar and inhibits tumor xenograft formation in immunodeficient mice [65]. More work is required to understand if downregulation of the Wnt pathway also inhibits differentiation in aRMS and sRMS tumors, as it does in eRMS.

In conclusion, downregulation of the Wnt pathway appears to play an important role in RMS tumorigenesis. Thus, activating the Wnt pathway in RMS tumors may be a promising future strategy for the treatment of both aRMS and eRMS.

### 3.3. Hedgehog and Rhabdomyosarcoma

 Clinical evidence supporting a role for Hedgehog signaling in RMS etiology is well established. Individuals with germline mutations in *PTCH1* (Gorlin syndrome) are susceptible to multiple malignancies such as basal cell carcinoma (BCC), medulloblastoma, and to a lesser extent RMS [66]. In parallel, mice that are heterozygous for Ptch1 demonstrate a phenotype similar to patients with Gorlin syndrome [67]; however, differences in mouse strain or background have a marked effect on susceptibility to RMS, which may explain the overall low frequency of RMS in Gorlin syndrome patients, when compared to BCC and medulloblastoma [68]. The role of *PTCH1* mutations in sporadic human RMS is less clear. Recent studies in human tumors that searched for mutations in either *PTCH1* or *SMO *have yielded conflicting results. For example, one study found that only one out of 14 RMS samples sequences demonstrated loss of heterozygosity (LOH) at the *PTCH1* locus and there were no point mutations or deletions found in the RMS samples tested [69]. In contrast, another group demonstrated that four out of 12 RMS tumors exhibited loss of 9q22 (containing *PTCH1*), suggesting that it may play a role in sporadic RMS [70]. In addition, a recent study found that 12 out of 34 fetal rhabdomyomas and eRMS lacked PTCH1 immunoreactivity and four of nine tumors examined had LOH at the *PTCH1 *locus [71].

Because eRMS is one of the three major tumor types that develop in *Ptch1^+/−^* mutant mice [72] and the other two tumor types (BCC and medulloblastoma) have shown response to SMO inhibition *in vivo *[73], it was assumed that targeting the Hedgehog pathway would have utility in eRMS. However, such an approach has proven challenging because even though *Ptch1^+/−^* mouse tumors depend on Smo for RMS initiation, the SMO inhibitor cyclopamine does not appear to suppress tumor growth *in vivo *[74]. Studies with human eRMS cell lines also failed to respond to cyclopamine *in vitro *and *in vivo*. However, Hedgehog signaling was still implicated in these same RMS cell lines because they responded to a novel GLI antagonist GANT 61 (Gli Antagonist 61) [23]. In addition, Gerber et al. demonstrated that overexpression of Gli1 inhibited differentiation of myogenic RMS cell lines [24]. In these studies, they showed that Gli1 and Gli2 repress the ability of MyoD to activate transcription and drive myogenic differentiation. Finally, a recent study observed that approximately 30% of fusion-negative RMSs contain an altered SHH pathway signature, consistent with activation of the pathway [75].

In contrast to eRMS, the Hedgehog pathway is not known to play a significant role in aRMS. In fact, recent studies on human tissue samples demonstrated not only that aRMS tumors had reduced levels of GLI1 transcript but that aRMS samples that harbored the 12q13-15 amplicon containing the *GLI1* gene did not lead to increased protein expression [76].

## 4. Drugs That Target the Notch, Wnt, and Hedgehog Embryonic Pathways

As discussed above, defects in the embryonic signaling pathways that control muscle development may underlie some of the aberrant proliferation and arrested differentiation in RMS. Developmental programs responsible for controlling self-renewal and differentiation in muscle progenitor cells, including Notch, WNT, and Hedgehog, may contribute to RMS tumorigenesis and are therefore attractive pathways for therapeutic targeting. Currently, several drugs targeting the Notch, WNT, or Hedgehog pathways are in pediatric (Table 2) and/or sarcoma (Table 3) clinical trials. Options for pharmacologic targeting of the Notch pathway include *γ*-secretase inhibitors, which block the proteolytic cleavage and subsequent activation of the Notch receptors [77]. While these drugs have been evaluated in clinical phase I and II trials, their side effect profile, including gastrointestinal toxicities, currently precludes broader evaluation. Further, this enzyme complex targets other substrates in addition to Notch receptor as evidenced by their initial development for treatment of Alzheimer's disease [78]. More direct inhibition of the Notch transcription factor complex, without the attendant gastrointestinal toxicity, has been achieved using hydrocarbon-stapled peptides, but this pharmacologic approach is still early in its preclinical development [79].

Regarding the Wnt pathway, most drugs being developed as cancer therapeutics are aimed at inhibiting the pathway [80, 81] since as described above when Wnt signaling is mutated in human cancer it is usually upregulated. These inhibitors appear to work at different levels of the Wnt pathway; for example, XAV939 inhibits Axin, while iCRT-3, 5, and 14 inhibit the *β*-catenin-TCF interaction [82, 83]. Recently, a phase I trial evaluating a chimeric humanized monoclonal antibody against FZD10 (Frizzled Family Receptor 10) has opened for patients with synovial sarcoma (Table 3), so that inhibiting the Wnt pathway at the level of a Frizzled receptor might also become a possibility. For RMS, current evidence suggests that one would need to activate the Wnt pathway. Such drugs include lithium chloride and lithium salts, which are known to activate the Wnt pathway through inhibition of GSK3 [84]. Underscoring the relative safety record of lithium salts, they are used to treat bipolar disorder not only in adulthood but during older childhood and adolescence [85], and even during pregnancy, with limited risk to the fetus despite equilibration across the placenta [86, 87]. Lithium chloride was also studied in adult patients with low-grade neuroendocrine tumors (NET), as GSK3*β* regulates growth and hormone production in NETs; however, it was ineffective at inhibiting NET growth [88]. In recent years, other drugs that activate the Wnt pathway have been developed. Maleimide derivatives SB216763 and SB415286 and indirubin analogs BIO and INO all selectively inhibit GSK3 [89, 90]. WAY-316606, a small-molecule inhibitor of SFRP1, was shown to stimulate bone formation in an *in vitro* model of osteoporosis [91].

Although cyclopamine does not appear to be effective for Ptch1-mutant RMS, it is possible that pharmacologic blockade of the Hedgehog pathway with other drugs will be a useful approach for treating these tumors. For example, other drugs that inhibit SMO have recently been developed including SANT-1, SANT-2, and GDC-0449 [92, 93] and drugs listed in Tables 2 and 3. Interestingly, a recent phase I trial demonstrated that GDC-0449 caused a partial or complete response in the majority of patients with BCC and medulloblastoma [73]. Whether these or other drugs that target other components of the Hedgehog pathway will be effective in treating RMS remains to be established.

In summary, there is enough evidence supporting a link between the Notch, Wnt, and Hedgehog embryonic pathways and RMS tumorigenesis to continue evaluating the blockade of these pathways in preclinical studies. But the optimism of these observations must be balanced by the feasibility of targeting these pathways in children. Therapies designed to target aberrant stem cell pathways involved in tumor development may also unwittingly inhibit normal, essential stem cell pathways required for normal growth and development into adulthood, a defining characteristic of the pediatric population.

## 5. Discussion

Embryonic cancers such as RMS are believed to result at least in part from defective developmental processes. As such, the genes and signaling pathways responsible for normal regulation of myogenic proliferation and differentiation remain vulnerable for transformation and may act as potential RMS oncogenes. It has been hypothesized that RMS may arise from muscle stem cells or, alternatively, differentiated muscle cells may undergo mutations that stimulate dedifferentiation and reactivate self-renewal. Regardless of the precise cell of origin, mutations in embryonic signaling pathways may function as primary events in tumorigenesis, or secondary, cooperating mutations. An improved understanding of the roles of embryonic signaling pathways in RMS tumorigenesis will allow for the identification of critical targets and promote the development of rational therapies.

The Notch, WNT, and Hedgehog pathways are regulators of myogenic lineage determination and maturation, and, accordingly, RMS may arise from disordered regulation of these normal developmental pathways. In this paper, we have summarized the known role of these signaling pathways during myogenesis and the current data supporting a link to rhabdomyosarcomagenesis. In summary, these embryonic signaling pathways are responsible for controlling the balance between undifferentiated muscle progenitor cells and nonproliferative differentiated myotubes. By favoring self-renewal versus a differentiated phenotype, muscle stem cells are at an increased risk for accumulating mutations necessary for oncogenesis; this is more likely to occur in cells that self-renew and do not exit the cell cycle or undergo apoptosis.

Rational drug targets are being identified and employed in RMS clinical trials (IGF1R inhibition, antiangiogenic approaches, and mTOR inhibition). However, progress has been slow for a variety of reasons. First, despite being the most common soft tissue sarcoma in childhood, RMS is still a rare disease. Therefore, there are relatively few patients available to enroll on early phase trials, and there are an increasing number of novel therapies with promising preclinical data competing for patient accrual. Second, many patients who are eligible for phase I studies do not choose to participate due to a history of heavy pretreatment and a general sense of “hopelessness” associated with “toxicity trials.” One strategy to overcome both of these obstacles is to open phase I trials of targeted therapeutics to patients who achieve remission but remain at high risk for disease relapse. This approach of investigating novel therapeutics as “maintenance therapy” would increase the number of patients available for enrollment in phase I trials and would also likely result in a higher accrual rate. A population of patients in remission would be healthier than patients with relapse, and they might be more likely to perceive hope with novel, single-agent therapies aimed at maintaining a first remission as opposed to inducing a second remission. In support of this approach, the most recent European Cooperative Sarcoma Group trial, CWS-96, demonstrated an improved survival for patients with metastatic soft tissue sarcomas treated with oral maintenance therapies compared to high-dose chemotherapy with a five-year overall survival of 52% versus 15%, respectively, [94]. While this study was a nonrandomized design and had relatively small numbers, the treatment groups had similar characteristics, and the differences were statistically significant suggesting that maintenance regimens are feasible and a promising strategy for the treatment of high risk RMS.

Based on the data presented in this paper, we conclude that the Notch, Wnt, and Hedgehog embryonic signaling pathways are important in RMS for the transformation of precursor cells, initiation of tumors, and maintenance of self-renewal properties. Given the persistently dismal cure rates for patients with high-risk RMS, an improved understanding of the role of these pathways in RMS may provide an opportunity to pharmacologically target these embryonic pathways and improve survival of patients with RMS.

## Figures and Tables

**Table 1:** Embryonic signaling pathways in rhabdomyosarcoma.

Embryonic pathway	Mechanism	Reference
Notch	(i) Promotes proliferation, suppresses differentiation, and avoids irreversible cell cycle arrest	[18]
	(ii) Promotes invasiveness and mobility	[19]
	(iii) Promotes proliferation and suppresses differentiation	[20]

WNT	(i) Suppresses differentiation and resists apoptosis	[21]
	(ii) Promotes proliferation and resists apoptosis	[22]

Hedgehog	(i) Promotes proliferation	[23]
	(ii) Suppresses differentiation	[24]

**Table 2:** Clinical trials evaluating drugs that target Notch, WNT, or Hedgehog signaling pathways in children.

ClinicalTrials.gov no.	Indication	Compound	Mechanism	Phase	Start date	Completion date	Sponsor/collaborator
Notch							

NCT00100152	Relapsed/refractory T cell ALL/lymphoma	MK0752	Gamma-secretase inhibitor	I	Jul 2005	Oct 2006	Merck
NCT01088763	Relapsed/refractory solid tumors, CNS tumors, lymphoma, T cell leukemia	RO4929097	Gamma-secretase inhibitor	I/II	Mar 2010	May 2011	Children's Oncology Group/NCI
NCT01236586	Relapsed/refractory solid tumors, CNS tumors, lymphoma, T cell leukemia	RO4929097	Gamma-secretase inhibitor	I/II	Oct 2010	Apr 2011	NCI/NIH CC

WNT							

Hedgehog							

NCT00822458	Recurrent/refractory medulloblastoma	GDC-0049	Smo small-molecule inhibitor	I	Jan 2009	Jul 2011	Pediatric brain tumor consortium/NCI
NCT01239316	Recurrent/refractory medulloblastoma	GDC-0449	Smo small-molecule inhibitor	II	Nov 2010	Ongoing	Pediatric brain tumor consortium/NCI
NCT01125800	Medulloblastoma, rhabdomyosarcoma, neuroblastoma, hepatoblastoma, high-grade glioma, astrocytoma	LDE225	Smo small-molecule inhibitor	I	Feb 2011	Ongoing	Novartis Pharmaceuticals

Obtained from clinicaltrials.gov website January 8, 2012.

**Table 3:** Clinical trials evaluating drugs that target Notch, WNT, or Hedgehog signaling pathways in sarcomas.

ClinicalTrials.gov no.	Indication	Compound	Mechanism	Phase	Start date	Completion date	Sponsor/collaborator
Notch							

NCT01154452	Advanced or metastatic sarcoma	RO4929097	Gamma-secretase inhibitor	I/II	Jun 2010	Nov 2010	Memorial Sloan Kettering Cancer Center/NCI
NCT01236586	Relapsed/refractory solid tumors, CNS tumors, lymphoma, T-cell leukemia	RO4929097	Gamma-secretase inhibitor	I/II	Oct 2010	Apr 2011	NCI/NIH CC

WNT							

NCT01469975	Synovial sarcoma	OTSA101	Chimeric humanized monoclonal antibody against FZD10	I	Dec 2011	Dec 2013	Centre Leon Berard/OncoTherapy Science, Inc.

Hedgehog							

NCT01154452	Advanced or metastatic sarcoma	GDC-0449	Smo small-molecule inhibitor	I/II	Jun 2010	Nov 2010	Memorial Sloan Kettering Cancer Center/NCI
NCT01267955	Advanced chondrosarcoma	GDC-0449	Smo small-molecule inhibitor	II	Dec 2010	Feb 2012	Institut Bergonié/NCI
NCT01310816	Metastatic or unresectable chondrosarcoma	IPI-296	Smo small-molecule inhibitor	II	Feb 2011	Sep 2015	Infinity Pharmaceuticals, Inc.
NCT01125800	Medulloblastoma, rhabdomyosarcoma, neuroblastoma, hepatoblastoma, high-grade glioma, astrocytoma	LDE225	Smo small-molecule inhibitor	I	Feb 2011	Ongoing	Novartis Pharmaceuticals

Obtained from clinicaltrials.gov website January 8, 2012.
